# *In silico* drug metabolism and pharmacokinetic profiles of natural products from medicinal plants in the Congo basin

**DOI:** 10.1186/2193-9616-1-12

**Published:** 2013-08-08

**Authors:** Fidele Ntie-Kang, Lydia L Lifongo, James A Mbah, Luc C Owono Owono, Eugene Megnassan, Luc Meva’a Mbaze, Philip N Judson, Wolfgang Sippl, Simon M N Efange

**Affiliations:** CEPAMOQ, Faculty of Science, University of Douala, P.O. Box 8580, Douala, Cameroon; Chemical and Bioactivity Information Centre, Department of Chemistry, Faculty of Science, University of Buea, P.O. Box 63, Buea, Cameroon; Department of Pharmaceutical Sciences, Martin-Luther University of Halle-Wittenberg, Wolfgang-Langenbeck Str. 4, 06120 Halle (Saale), Germany; Department of Chemistry, Faculty of Science, University of Buea, P.O. Box 63, Buea, Cameroon; Laboratory for Simulations and Biomolecular Physics, Advanced Teachers Training College, University of Yaoundé, I, P.O. Box 47, Yaoundé, Cameroon; Laboratory of Fundamental and Applied Physics, University of Abobo-Adjame, Abidjan, 02 BP 801 Cote d’Ivoire; Department of Chemistry, Faculty of Science, University of Douala, P. O. Box 24157, Douala, Cameroon; Chemical and Bioactivity Information Centre, 22-23 Blenheim Terrace, Woodhouse Lane, Leeds LS2 9HD UK

**Keywords:** ADMET, Drug discovery, Descriptors, *In silico*, Medicinal plants, Natural products

## Abstract

**Purpose:**

Drug metabolism and pharmacokinetics (DMPK) assessment has come to occupy a place of interest during the early stages of drug discovery today. The use of computer modelling to predict the DMPK and toxicity properties of a natural product library derived from medicinal plants from Central Africa (named ConMedNP). Material from some of the plant sources are currently employed in African Traditional Medicine.

**Methods:**

Computer-based methods are slowly gaining ground in this area and are often used as preliminary criteria for the elimination of compounds likely to present uninteresting pharmacokinetic profiles and unacceptable levels of toxicity from the list of potential drug candidates, hence cutting down the cost of discovery of a drug.

In the present study, we present an *in silico* assessment of the DMPK and toxicity profile of a natural product library containing ~3,200 compounds, derived from 379 species of medicinal plants from 10 countries in the Congo Basin forests and savannas, which have been published in the literature. In this analysis, we have used 46 computed physico-chemical properties or molecular descriptors to predict the absorption, distribution, metabolism and elimination and toxicity (ADMET) of the compounds.

**Results:**

This survey demonstrated that about 45% of the compounds within the ConMedNP compound library are compliant, having properties which fall within the range of ADME properties of 95% of currently known drugs, while about 69% of the compounds have ≤ 2 violations. Moreover, about 73% of the compounds within the corresponding “drug-like” subset showed compliance.

**Conclusions:**

In addition to the verified levels of “drug-likeness”, diversity and the wide range of measured biological activities, the compounds from medicinal plants in Central Africa show interesting DMPK profiles and hence could represent an important starting point for hit/lead discovery.

**Electronic supplementary material:**

The online version of this article (doi:10.1186/2193-9616-1-12) contains supplementary material, which is available to authorized users.

## Background

Natural products (NPs) have always played an important role in drug discovery until today (Li and Vederas 2009; Chin et al. 2006; Newman 2008; Harvey 2008; Koehn and Carter 2005). This is because they both serve as active principles in drugs and as templates for the synthesis of new drugs (Newman 2008; Efange 2002). Additionally, a good proportion of drugs which have been approved for clinical trials, are either NPs or their analogues (Butler 2005). What makes NPs unique is that they are often rich in stereogenic centres and cover segments of chemical space which are typically not occupied by a majority of their synthetic counterparts (Wetzel et al. 2007; Grabowski et al. 2008). Moreover, NPs generally contain more oxygen atoms and less aromatic atoms on average, when compared with “drug-like” molecules (Grabowski and Schneider 2007). They sometimes fail the test for “drug-likeness” due to the fact that they are often bulkier than synthetic drugs (Quinn et al. 2008).

The fact that more and more drugs fail to enter the market as a result of poor pharmacokinetic profiles, has necessitated the inclusion of pharmacokinetic considerations at earlier stages of drug discovery programs (Hodgson 2001; Navia and Chaturvedi 1996). This requires the search for lead compounds which can be easily orally absorbed, easily transported to their desired site of action, not easily metabolised into toxic products before reaching the targeted site of action and easily eliminated from the body before accumulating in sufficient amounts that may produce adverse side effects. The sum of the above mentioned properties is often referred to as ADME (absorption, distribution, metabolism and elimination) properties, or better still ADMET, ADME/T or ADMETox (when toxicity assessment is included).

Computer-based methods have been employed in the prediction of ADMET properties of drug leads at early stages of drug discovery and such approaches are becoming increasingly popular (Lipinski et al. 1997; Lombardo et al. 2003; Gleeson et al. 2011). The rationale behind *in silico* approaches are the relatively lower cost and the time factor involved, when compared to standard experimental approaches for ADMET profiling (DiMasi et al. 2003; Darvas et al. 2002). As an example, it takes a minute in an *in silico* model to screen 20,000 molecules, but takes 20 weeks in the “wet” laboratory to do the same exercise (Hodgson 2001). Due to the accumulated ADMET data in the late 1990s, many pharmaceutical companies are now using computational models that, in some cases, are replacing the “wet” screens (Hodgson 2001). This paradigm shift has therefore spurred up the development of several theoretical methods for the prediction of ADMET parameters. A host of these theoretical models have been implemented in a number of software programs currently available for drug discovery protocols (OCHEM platform 2009; Lhasa 2010; Schrodinger 2011a; Cruciani et al. 2000), even though some of the predictions are often disappointing (Tetko et al. 2006). The software tools currently used to predict the ADMET properties of potential drug candidates often make use of quantitative structure-activity relationships, QSAR (Tetko et al. 2006; Hansch et al. 2004) or knowledge-base methods (Greene et al. 1999; Button et al. 2003; Cronin 2003). A promising lead compound may therefore be defined as one which combines potency with an admirable ADMET profile (commonly referred to as a compound’s CV). As such, compounds with uninteresting predicted ADMET profiles may be completely dismissed from the list of potential drug candidates (even if these prove to be highly potent). Otherwise, the DMPK properties are “fine-tuned” in order to improve their chances of making it to clinical trials (Hou and Wang 2008). This may explain why the “graveyard” of very highly potent compounds which do not make it to clinical trials keeps filling up, to the extent that experts in drug discovery are often faced with the challenge of either resorting to new lead compounds or “resurrecting” some buried leads with the view of “fine-tuning” their DMPK properties.

A natural product compound database built on information collected from several literature sources on medicinal plants from Central African countries, currently used in ATM, has been recently developed at our laboratory. The plants had been harvested from 10 countries (Burundi, Cameroon, Central African Republic, Chad, Congo, Equatorial Guinea, Gabon, the Democratic Republic of Congo, Rwanda and the Republic of São Tomé and Príncipe). This NP library currently contains ~3,200 compounds and preliminary analyses have proven the dataset to be sufficiently “drug-like” and diverse to be employed in lead discovery programs (Ntie-Kang et al. 2013a; Ntie-Kang et al.: ConMedNP: a natural product library from Central African medicinal plants for drug discovery. RSC Adv, submitted). Additional arguments in favour of the use of this database are the wide range of the previously observed biological activities of the compounds and the wide range of ailments being treated by traditional medicine with the help of the herbs from which the compounds have been derived (Ntie-Kang et al. 2013a, b; Zofou et al. 2013; Ntie-Kang et al.: ConMedNP: a natural product library from Central African medicinal plants for drug discovery. RSC Adv, submitted).

Since numerous drugs and many more lead compounds fail due to adverse pharmacokinetic properties at a late stage of pharmaceutical development (Darvas et al. 2002), it has become important to incorporate ADME properties’ prediction into the lead compound selection early enough, by means of molecular descriptors. A molecular descriptor may be defined as a structural or physico-chemical property of a molecule or part of a molecule, for example logarithm of the *n*-octanol/water partition coefficient (log *P*), the molar weight (MW) and the total polar surface area (TPSA). A number of relevant molecular properties (descriptors) are often used to help in the assessment of the DMPK properties of potential drug leads. In this paper, an attempt has been made to carry out an *in silico* assessment of the ADMET profile of this dataset. A number of computed molecular descriptors, currently implemented in a wide range of software, have been used as indicators of the pharmacokinetic properties of a large proportion of currently known drugs.

## Methods

### Data sources and generation of 3D structures

The plant sources, geographical collection sites, chemical structures of pure compounds as well as their measured biological activities, were retrieved from literature sources and have been previously described (Ntie-Kang et al. 2013b; Zofou et al. 2013). The 3D structures of the compounds had been sketched and energy minimisation subsequently carried out using a previously described protocol (Ntie-Kang et al. 2013a).

### Initial treatment of chemical structures and calculation of ADMET-related descriptors

The 3,179 low energy 3D chemical structures in the ConMedNP library were saved in .mol2 format and initially treated with LigPrep (Schrödinger 2011a). This implementation was carried out with the graphical user interface (GUI) of the Maestro software package (Schrödinger 2011b), using the Optimized Potentials for Liquid Simulations (OPLS) forcefield (Shivakumar et al. 2010; Jorgensen et al. 1996; Jorgensen et al. 1988). Protonation states at biologically relevant pH were correctly assigned (group I metals in simple salts were disconnected, strong acids were deprotonated, strong bases protonated, while topological duplicates and explicit hydrogens were added). All molecular modelling was carried out on a Linux workstation with a 3.5 GHz Intel Core2 Duo processor. A set of ADMET-related properties (a total of 46 molecular descriptors) were calculated by using the QikProp program (Schrödinger 2011c) running in normal mode. QikProp generates physically relevant descriptors, and uses them to perform ADMET predictions. An overall ADME-compliance score – drug-likeness parameter (indicated by #stars), was used to assess the pharmacokinetic profiles of the compounds within the ConMedNP library. The #stars parameter indicates the number of property descriptors computed by QikProp that fall outside the optimum range of values for 95% of known drugs. The methods implemented were developed by Jorgensen and Duffy (Jorgensen and Duffy 2002; Duffy and Jorgensen 2000; Jorgensen and Duffy 2000). Some of the computed ADMET descriptors are shown in Table 1, along with their recommended ranges for 95% of known drugs.

**Table 1:** **Selected computed ADMET-related descriptors and their recommended ranges for 95% of known drugs**

Property	Description	Recommended range
S_*mol*_	the total solvent-accessible molecular surface, in Å^2^ (probe radius 1.4 Å)	300 to 1000 Å^2^
S_*mol,hfob*_	the hydrophobic portion of the solvent-accessible molecular surface, in Å^2^ (probe radius 1.4 Å)	0 to 750 Å^2^
V_*mol*_	the total volume of molecule enclosed by solvent-accessible molecular surface, in Å^3^ (probe radius 1.4 Å)	500 to 2000 Å^3^
log S_*wat*_	the logarithm of aqueous solubility (Jorgensen and Duffy 2002; Jorgensen and Duffy 2000)	−6.0 to 0.5
log *K*_*HSA*_	the logarithm of predicted binding constant to human serum albumin (Colmenarejo 2001)	−1.5 to 1.2
log *B/B*	the logarithm of predicted blood/brain barrier partition coefficient (Luco 1999; Kelder et al. 1999; Ajay et al. 1999)	−3.0 to 1.0
*BIP*_*caco*–2_	the predicted apparent Caco-2 cell membrane permeability, in nm s^-1^ (in Boehringer–Ingelheim scale, Yazdanian et al. Yazdanian et al. 1998; Irvine et al. 1999; Stenberg 2001)	< 5 low, > 100 high
*MDCK*	the predicted apparent Madin-Darby canine kidney cell permeability in nm s^-1^ (Irvine et al. 1999)	< 25 poor, > 500 great
Ind_coh_	the index of cohesion interaction in solids, calculated from the number of hydrogen bond acceptors (HBA), donors (HBD) and the surface area accessible to the solvent, SASA (*S*_*mol*_) by the relation (Jorgensen and Duffy 2000)	0.0 to 0.05
Glob	the globularity descriptor, Glob = (4*πr* ^2^)/*S*_*mol*_, where *r* is the radius of the sphere whose volume is equal to the molecular volume	0.75 to 0.95
*QP*_*polrz*_	the predicted polarizability	13.0 to 70.0
log *HERG*	the predicted IC_50_ value for blockage of HERG K^+^ channels, (Cavalli et al. 2002; De Ponti et al. 2001)	concern < −5
log *K*_p_	the predicted skin permeability (Potts and Guy 1992; Potts and Guy 1995)	−8.0 to −1.0
*#metab*	the number of likely metabolic reactions	1 to 8

## Results and discussion

### Overall DMPK compliance of the ConMedNP library

**Figure 1:**
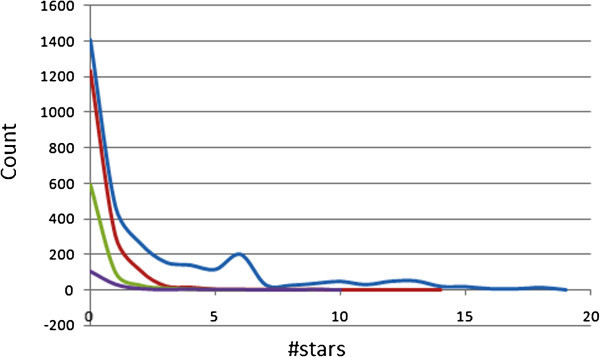
**Distribution curves for #stars within the ConMedNP library, along with the standard “drug-like”, “lead-like” and “fragment-like” subsets.** Blue = ConMedNP library, red = “drug-like” subset, green = “lead-like” subset and violet = “fragment-like” subset.

The 24 most relevant molecular descriptors calculated by QikProp are used to determine the #star parameter (Schrödinger 2011d). A plot of the #stars parameter (on *x*-axis) against the corresponding counts (on *y*-axis) in the ConMedNP is plotted within the same set of axes with those of the “drug-like”, “lead-like”, and “fragment-like” standard subsets (Figure 1). The criteria for the respective standard subsets were defined as (MW < 500; log *P* < 5; HBD ≤ 5; HBA ≤ 10) (Lipinski et al. 1997), (150 ≤ MW ≤ 350; log *P* ≤ 4; HBD ≤ 3; HBA ≤ 6) (Teague et al. 1999; Oprea 2002; Schneider 2002) and (MW ≤ 250; -2 ≤ log *P* ≤ 3; HBD < 3; HBA < 6; NRB < 3) (Verdonk et al. 2003). The ADMET descriptors for some 67 compounds in the total library were not computed by QikProp, probably due to some technical details related to the working of the software which was beyond our notice. Of the remaining 3,112 compounds, 45.31% showed #star = 0, while 68.93% had #star ≤ 2. Among the 1,696 compounds of the “drug-like” subset whose pharmacokinetic properties were predicted, 72.52% had pharmacokinetic descriptors within the acceptable range for 95% of known drugs, while 96.88% showed #stars ≤ 2. The “lead-like” and “fragment-like” subsets were respectively 80.68% and 65.58% compliant for all of the 24 most relevant computed descriptors. The mean values for 19 selected computed descriptors have been shown in Table 2 for all 4 compound libraries, while percentage compliances for 14 selected parameters are shown in Table 3. The mean values were used to assess the probability of finding drug leads within the ConMedNP compound library.

**Table 2:** **Summary of mean pharmacokinetic property distributions of the total ConMedNP library in comparison with the various subsets**

Library name	^***a***^Lib. size	^***b***^No. compl.	^***c***^MW (Da)	^***d***^Log***P***	^***e***^HBA	^***f***^HBD	^***g***^NRB
**Total library**	3,118	1,410	421.72	3.86	6.54	2.03	7.46
**Drug-like**	1,696	1,230	326.14	2.76	5.13	1.43	4.47
**Lead-like**	730	589	269.55	2.24	4.26	1.17	3.23
**Fragment-like**	154	101	192.98	1.33	3.58	0.89	2.17
	^***h***^ **LogB/B**	^***i***^ **BIP**_**caco-2**_ **(nm s** ^**-1**^ **)**	^***j***^ ***S***_**mol**_ **(Å** ^**2**^ **)**	^***k***^ ***S***_**mol,hfob**_ **(Å** ^**2**^ **)**	^***l***^ ***V***_**mol**_ **(Å** ^**3**^ **)**	^***m***^ **Log** ***S***_***wat***_ **(S in mol L** ^**-1**^ **)**	^***n***^ **Log** ***K***_***HSA***_
**Total library**	−1.23	1322.58	696.98	420.94	1314.78	−5.16	0.52
**Drug-like**	−0.77	1195.51	564.70	274.17	1014.47	−3.78	0.13
**Lead-like**	−0.57	1233.11	494.87	204.27	856.93	−3.04	−0.07
**Fragment-like**	−0.47	1178.35	393.88	111.25	640.36	−1.78	−0.48
	^**o**^ **MDCK**	^***p***^ **Ind**_**coh**_	^***q***^ **Glob**	^***r***^ **QP**_**polrz**_ **(Å** ^**3**^ **)**	^***s***^ **LogHERG**	^***t***^ **Log** ***K***_***p***_	^***u***^ **# metab**
**Total library**	765.31	0.013	0.84	42.99	−4.60	−2.99	5.53
**Drug-like**	689.00	0.009	0.87	33.17	−4.39	−2.91	4.55
**Lead-like**	717.25	0.008	0.88	27.75	−4.17	−2.85	3.40
**Fragment-like**	682.72	0.007	0.91	19.67	−3.46	−2.81	1.90

^*a*^Size or number of compounds in library; ^*b*^Number of compounds with #star = 0; ^*c*^Molar weight (range for 95% of drugs: 130–725 Da); ^*d*^Logarithm of partitioning coefficient between *n*-octanol and water phases (range for 95% of drugs: -2 to 6.5); ^*e*^Number of hydrogen bonds accepted by the molecule (range for 95% of drugs: 2–20); ^*f*^Number of hydrogen bonds donated by the molecule (range for 95% of drugs: 0–6).; ^*g*^Number of rotatable bonds (range for 95% of drugs: 0–15); ^*h*^Logarithm of predicted blood/brain barrier partition coefficient (range for 95% of drugs: -3.0 to 1.0); ^*i*^Predicted apparent Caco-2 cell membrane permeability in Boehringer–Ingelheim scale, in nm/s (range for 95% of drugs: < 5 low, > 500 high); ^*j*^Total solvent-accessible molecular surface, in Å^2^ (probe radius 1.4 Å) (range for 95% of drugs: 300–1000 Å^2^); ^*k*^Hydrophobic portion of the solvent-accessible molecular surface, in Å^2^ (probe radius 1.4 Å) (range for 95% of drugs: 0–750 (Å^2^); ^*l*^Total volume of molecule enclosed by solvent-accessible molecular surface, in Å^3^ (probe radius 1.4 Å) (range for 95% of drugs: 500–2000 Å^3^); ^*m*^Logarithm of aqueous solubility (range for 95% of drugs: -6.0 to 0.5); ^*n*^Logarithm of predicted binding constant to human serum albumin (range for 95% of drugs: -1.5 to 1.5); ^*o*^Predicted apparent MDCK cell permeability in nm/sec (< 25 poor, > 500 great); ^*p*^Index of cohesion interaction in solids (0.0 to 0.05 for 95% of drugs); ^*q*^Globularity descriptor (0.75 to 0.95 for 95% of drugs); ^*r*^Predicted polarizability (13.0 to 70.0 for 95% of drugs); ^*s*^Predicted IC_50_ value for blockage of HERG K^+^ channels (concern < −5); ^*t*^Predicted skin permeability (−8.0 to −1.0 for 95% of drugs); ^*u*^Number of likely metabolic reactions (range for 95% of drugs: 1–8).

**Table 3:** **Summary of percentage compliances of selected ADMET-related descriptors of the total ConMedNP library in comparison with the various subsets**

Library name	^*^LogB/B	^*^BIP_caco-2_(nm s^-1^)	^*^ ***S***_mol_(Å^2^)	^*^ ***S***_mol,hfob_(Å^2^)	^*^ ***V***_mol_(Å^3^)	^*^Log***S***_***wat***_(S in mol L^-1^)	^*^Log***K***_***HSA***_
**Total library**	88.53	37.28	90.36	91.32	91.03	72.46	81.01
**Drug-like**	99.35	43.99	99.35	99.82	98.99	90.39	99.35
**Lead-like**	99.72	53.70	99.72	100.00	99.72	99.31	99.59
**Fragment-like**	100.00	33.11	95.45	100.00	92.21	97.40	98.05
	^*^ **MDCK**	^*^ **Ind**_**coh**_	^*^ **Glob**	^***a***^ **ro3**	^*^ **LogHERG**	^*^ **Log** ***K***_***p***_	^*^ **# metab**
**Total library**	47.14	94.92	89.88	43.57	58.35	92.09	81.52
**Drug-like**	59.61	99.14	97.70	73.52	63.62	95.93	91.89
**Lead-like**	59.86	100.00	97.81	93.56	76.03	97.53	96.44
**Fragment-like**	63.33	100.00	92.21	100.00	100.00	98.05	92.21

^*^Descriptors are defined in Tables 1 and 2; ^*a*^percentage compliance to Jorgensen’s Rule of Three.

### Bioavailability prediction

**Figure 2:**
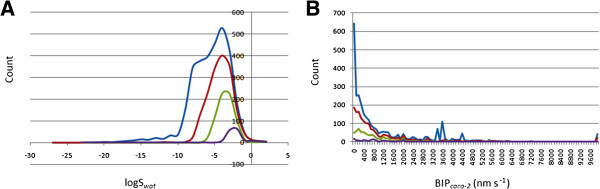
**Distribution curves for compliance to Jorgensen’s “Rule of Three”. (A)** calculated logS_wat_ against count, **(B)** predicted BIP_*caco*–2_ against count. Colour codes are as defined in Figure 1.

**Figure 3:**
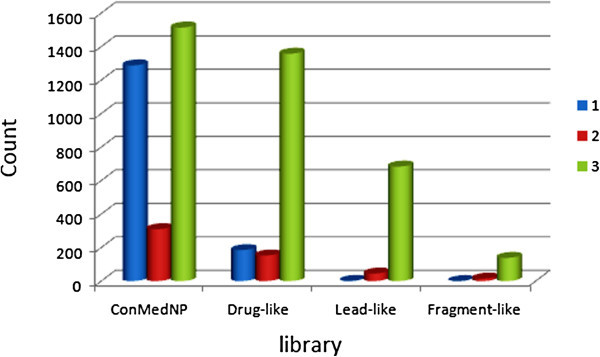
**Histograms showing the distribution of human oral absorption predictions.**

The bioavailability of a compound depends on the processes of absorption and liver first-pass metabolism (Van de Waterbeemd and Gifford 2003). Absorption in turn depends on the solubility and permeability of the compound, as well as interactions with transporters and metabolizing enzymes in the gut wall. The computed parameters used to assess oral absorption are the predicted aqueous solubility, logS_*wat*_*,* the conformation-independent predicted aqueous solubility, CI logS_*wat*_, the predicted qualitative human oral absorption, the predicted % human oral absorption and compliance to Jorgensen’s famous “Rule of Three” (ro3). The solubility calculation procedure implemented depends on the similarity property space between the given molecule and its most similar analogue within the experimental training set used to develop the model implemented in QikProp, i.e., if the similarity is < 0.9, then the QikProp predicted value is taken, otherwise, the predicted property, *P*_*pred*_, is adjusted such that:1

where *S* is the similarity, and *P*_*exp*_ and *P*_*QP*_ are the respective experimental and QikProp predictions for the most similar molecule within the training set. In equation (1), if *S* = 1, then the predicted property is equal to the measured experimental property of the training set compound. According to Jorgensen’s ro3, if a compound complies to all or some of the rules (logS_*wat*_ > −5.7, BIP_*caco*–2_ > 22 nm/s and # Primary Metabolites < 7), then it is more likely to be orally available. The distribution curves for two of the three determinants for the ro3 (logS_*wat*_ and BIP_*caco*–2_) are shown in Figure 2. In general 43.57% of the ConMedNP library was compliant to the ro3, while the respective % compliances for the various subsets were 73.52%, 93.56% and 100% for the “drug-like”, “lead-like” and “fragment-like” libraries. Among the individual computed parameters, the most remarkable was logS_*wat*_. This was met by 72.46% of the compounds within the ConMedNP library, while this property showed a Gaussian distribution for the “drug-like” and “lead-like” subsets. Only 37.28% of the compounds fell within the respected range for the BIP_*caco*–2_ criterion. The predicted apparent Caco-2 cell permeability, BIP_*caco*–2_ (in nm s^-1^), model the permeability of the gut-blood barrier (for non-active transport), even though this parameter is not often correctly predicted computationally (Veber et al. 2002). The histograms of the predicted qualitative human oral absorption parameter (in the scale 1 = low, 2 = medium and 3 = high) are shown in Figure 3. It was observed 48.65% of the compounds in ConMedNP were predicted to have high human oral absorption. The predicted % human oral absorption (on 0 to 100% scale) shows a similar trend, 42.09% of the compounds being predicted to be absorbed at 100% and 57.81% of the compounds predicted to be absorbed at > 90%.

A molecule’s size, as well as its capacity to make hydrogen bonds, its overall lipophilicity and its shape and flexibility are important properties to consider when determining permeability. Molecular flexibility has been seen as a parameter which is dependent on the number of rotatable bonds (NRB), a property which influences bioavailability in rats (Veber et al. 2002). The distribution of the NRB for this dataset has been discussed in detail elsewhere (Ntie-Kang et al. ConMedNP: a natural product library from Central African medicinal plants for drug discovery. RSC Adv, submitted) and the results reveal that the compounds within the ConMedNP library show some degree of conformational flexibility, the peak value for the NRB being between 1 and 2, while the average value is 7.46 (Table 2).

### Prediction of blood–brain barrier (BBB) penetration

**Figure 4:**
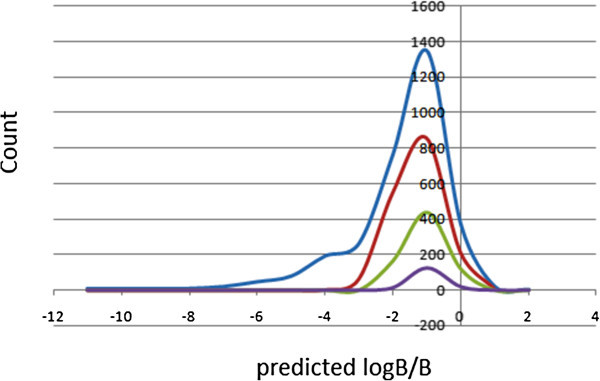
**Plot of the physico-chemical descriptor used to predict BBB penetration.** Predicted log *B/B* against count. The *x*-axis label is the lower limit of binned data, e.g. 0 is equivalent to 0.0 to 1.0. Colour codes are as defined in Figure 1.

Too polar drugs do not cross the BBB. The blood/brain partition coefficients (logB/B) were computed and used as a predictor for access to the central nervous system (CNS). The predicted CNS activity was computed on a −2 (inactive) to +2 (active) scale and showed that only 2.47% of the compounds in ConMedNP could be active in the CNS (predicted CNS activity > 1). A distribution of logB/B (Figure 4) shows a right-slanted Gaussian-shaped curve with a peak at −0.5 logB/B units (the same for all the standard subsets), with 88.53% of the compounds in ConMedNP falling within the recommended range for the predicted brain/blood partition coefficient (−3.0 to 1.2). Madin-Darby canine kidney (MDCK) monolayers, are widely used to make oral absorption estimates, the reason being that these cells also express transporter proteins, but only express very low levels of metabolizing enzymes (Veber et al. 2002). They are also used as an additional criterion to predict BBB penetration. Thus, our calculated apparent MDCK cell permeability could be considered to be a good mimic for the BBB (for non-active transport). It was estimated that only about 47% of the compounds had apparent MDCK cell permeabilities which fall within the recommended range of 25–500 nm s^-1^ for 95% of known drugs. This situation knew improvements in the “drug-like” and “lead-like” subsets (~60% for both subsets).

### Prediction of dermal penetration

**Figure 5:**
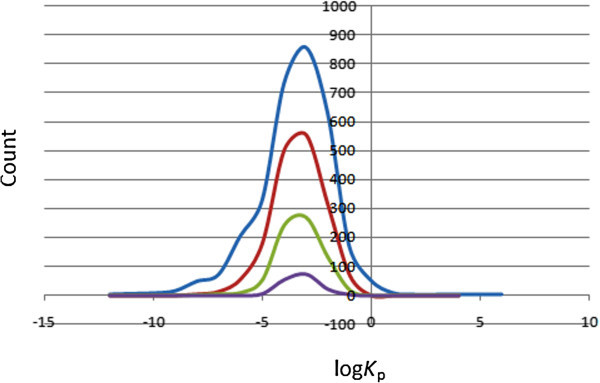
**Distribution curves for the predicted skin penetration parameter.** Colour codes are as defined in Figure 1.

This factor is important for drugs administered through the skin. The distribution of computed skin permeability parameter, log *K*_p_, showed smooth Gaussian-shaped graphs centred at −2.5 log *K*_P_ units for all 4 datasets (Figure 5), with ~92% of the compounds in the dataset falling within the recommended range for 95% of known drugs. The predicted maximum transdermal transport rates, *J*_*m*_ (in μ cm^-2^ hr^-1^), were computed from the aqueous solubility (S_*wat*_) and skin permeability (*K*_p_), using the relation (2):2

This parameter showed variations from 0 to 1603 μ cm^-2^ hr^-1^, with only about 1.38% of the compounds in ConMedNP having predicted value of *J*_*m*_ > 100 μ cm^-2^ hr^-1^.

### Prediction of plasma-protein binding

**Figure 6:**
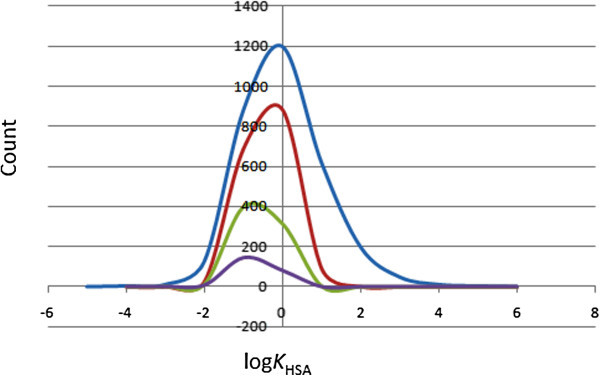
**Distribution curves for predicted plasma-protein binding.** Colour codes are as defined in Figure 1.

The efficiency of a drug may be affected by the degree to which it binds to the proteins within blood plasma. It is noteworthy that binding of drugs to plasma proteins (like human serum albumin, lipoprotein, glycoprotein, α, ββ and γ globulins) greatly reduces the quantity of the drug in general blood circulation and hence the less bound a drug is, the more efficiently it can traverse cell membranes or diffuse. The predicted plasma-protein binding has been estimated by the prediction of binding to human serum albumin; the log *K*_HSA_ parameter (recommended range is −1.5 to 1.5 for 95% of known drugs). Figure 6 shows the variation of this calculated parameter within the ConMedNP dataset, as well as for the standard subsets. This equally gave smooth Gaussian-shaped curves centred on −0.5 log *K*_HSA_ units for the total and “drug-like” libraries and −1.5 log *K*_HSA_ units for the “lead-like” and “fragment-like” datasets. In addition, our calculations reveal that > 81% of the compounds within the ConMedNP library are compliant to this parameter, indicating that a majority of the compounds are likely to circulate freely within the blood stream and hence have access to the target site.

### Metabolism prediction

**Figure 7:**
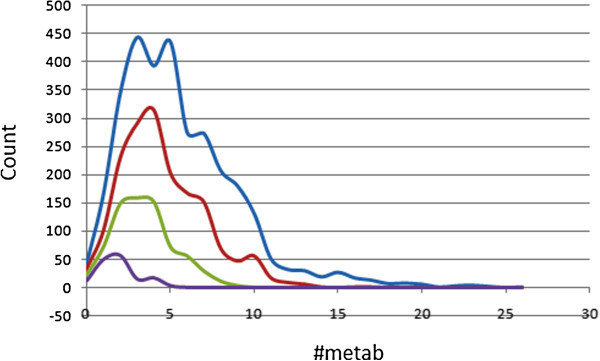
**Graphs showing the distribution of the predicted number of metabolic reactions for compounds in ConMedNP.** Colour codes are as defined in Figure 1.

An estimated number of possible metabolic reactions has also been predicted by QikProp and used to determine whether the molecules can easily gain access to the target site after entering the blood stream. The average estimated number of possible metabolic reactions for the ConMedNP library is between 5 and 6, while those of the standard subsets are respectively between 4 and 5, between 3 and 4 and between 1 and 2 for the “drug-like”, “lead-like” and “fragment-like” libraries (Table 2). Even though some of the compounds are likely to undergo as many as up to 26 metabolic reactions due to the complexity of some of the plant secondary metabolites within the database (Figure 7), ~81% of the compounds are predicted to undergo the recommended number of metabolic steps (1 to 8 reactions), with the situation improving to ~92% and almost 100% in the “drug-like” and “lead-like” subsets respectively. From Figure 7, it can be observed that the total and “lead-like” libraries show peak values at 3 metabolic steps, while the drug-like subset rather shows a peak at 4 metabolic steps and the “fragment-like” subsets peaks at 2 predicted metabolic reactions.

### Prediction of blockage of human ether-a-go-go-related gene potassium (HERG K^+^) channel

**Figure 8:**
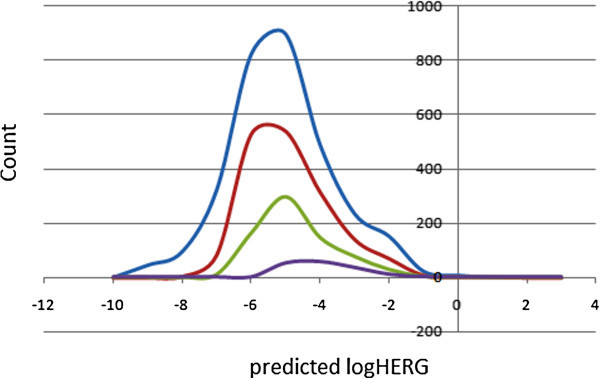
**A plot of predicted logHERG values for ConMedNP and standard subsets.** Colour codes are as defined in Figure 1.

Human ether-a-go-go related gene (HERG) encodes a potassium ion (K^+^) channel that is implicated in the fatal arrhythmia known as *torsade de pointes* or the long QT syndrome (Hedley et al. 2009). The HERG K^+^ channel, which is best known for its contribution to the electrical activity of the heart that coordinates the heart’s beating, appears to be the molecular target responsible for the cardiac toxicity of a wide range of therapeutic drugs (Vandenberg 2001). HERG has also been associated with modulating the functions of some cells of the nervous system and with establishing and maintaining cancer-like features in leukemic cells (Chiesa et al. 1997). Thus, HERG K^+^ channel blockers are potentially toxic and the predicted IC_50_ values often provide reasonable predictions for cardiac toxicity of drugs in the early stages of drug discovery (Aronov 2005). In this work, the estimated or predicted IC_50_ values for blockage of this channel have been used to model the process *in silico*. The recommended range for predicted log IC_50_ values for blockage of HERG K^+^ channels (logHERG) is > −5. A distribution curve for the variation of the predicted logHERG is shown in Figure 8, which is a left-slanted Gaussian-shaped curve, with a peak at −5.5 logHERG units for the total library, as well as for the “drug-like” and “lead-like” subsets. It was observed that in general, this parameter is predicted to fall within the recommended range for about 58% of the compounds within the ConMedNP dataset, ~64% for the “drug-like” subset and ~76% for the “lead-like” subset.

### Usefulness of the compound library

The usefulness of the ConMedNP database in lead generation has been exemplified with the docking and pharmacophore-based screening for potential inhibitors of a validated anti-malarial drug target in our laboratory, and the results will be published in a subsequent paper. It is important to mention that virtual screening results could provide insight and direct natural product chemists to search for theoretically active principles with attractive ADMET profiles, which have been previously isolated, but not tested for activity against specified drug targets (if samples are absent). This “resurrection” process could prove to be a better procedure for lead search than the random screening, which is a common practice in our African laboratories. This dataset is constantly being updated; meanwhile a MySQL platform to facilitate the searching of this database and ordering of compound samples is under development within our group and will also be published subsequently. However, 3D structures of the compounds, as well as their physico-chemical properties that were used to evaluate the DMPK profile, can be freely downloaded as additional files accompanying this publication (Additional files 1, 2, 3 and 4). In addition, information about compound sample availability can be obtained on request from the authors of this paper or from the pan-African Natural Products Library (p-ANAPL) project (Chibale et al. 2012; p-ANAPL 2013).

## Conclusions

Modern drug discovery programs usually involve the search for small molecule leads with attractive pharmacokinetic profiles. The presence of such within the ConMedNP library is of major importance and therefore renders the database attractive, in addition to the already known properties – “drug-like”, “lead-like”, “fragment-like” and diverse. This is an indication that the 3D structures of naturally occurring compounds within ConMedNP could be a good starting point for docking, neural networking and pharmacophore-based virtual screening campaigns, thus rendering ConMedNP a useful asset for the drug discovery community.

## Availability and requirements

3D structures of the compounds, as well as their physico-chemical properties that were used to evaluate the DMPK profile of the ConMedNP library, can be freely downloaded (for non commercial use) as additional files which accompany this publication (Additional files 1, 2, 3 and 4). Physical samples for testing are available at the various research laboratories in Central Africa in varying quantities. Questions regarding the available of compound samples could be addressed directly to the authors of this paper. Otherwise samples could be obtainable from the p-ANAPL consortium, which has a mandate to collect samples of NPs from the entire continent of Africa and make them available for biological screening. This network is being set up under the auspices of the Network for Analytical and Bioassay Services in Africa (NABSA) (Chibale et al. 2012; p- ANAPL 2013).

## Authors’ informations

WS and SMNE are professors of medicinal chemistry with an interest in CADD, while SMNE also focuses organic synthesis and on natural product leads from Cameroonian medicinal plants. LMM and JAM are natural product chemists actively involved in the isolation and characterization of secondary metabolites from Cameroonian medicinal plants. LLL holds a PhD in environmental science and manages a Chemical and Bioactivity Information Centre (CBIC) with a focus on developing databases for information from medicinal herbs in Africa. PNJ is a retired research officer of Lhasa Ltd who currently leads the CBIC branch in Leeds, UK. FNK is a PhD student working on CADD under the joint supervision of LCOO and EM.

## Electronic supplementary material

Additional file 1: **3D structures of compounds currently included in ConMedNP with calculated pharmacokinetic descriptors.** (SDF 18 MB)

Additional file 2: **3D structures of the “drug-like” subset with calculated pharmacokinetic descriptors.** (SDF 8 MB)

Additional file 3: **3D structures of the “lead-like” subset with calculated pharmacokinetic descriptors.** (SDF 3 MB)

Additional file 4: **3D structures of the “fragment-like” subset with calculated pharmacokinetic descriptors.** (SDF 484 KB)

## Abbreviations

3D: Three dimensional
ADME/T: Absorption, distribution, metabolism, excretion, and toxicity
CADD: Computer-aided drug design
ConMedNP: Congo basin medicinal plant and natural products database
DMPK: Drug metabolism and pharmacokinetics
log P: Logarithm of the octan-1-ol/water partition coefficient
MDCK: Madin-Darby canine kidney
MW: Molar weight
NABSA: Network for analytical and bioassay services in Africa
NP: Natural product
NRB: Number of rotatable bonds
OPLS: Optimized potentials for liquid simulations
p-ANAPL: pan-African Natural Products Library
SASA: Solvent accessible surface area
TPSA: Total polar surface area
VS: Virtual screening.
